# Legislation for Midwives

**Published:** 1900-07-14

**Authors:** 


### EDITOR'S LETTER-BOX.

[Our Correspondents are reminded that prolixity is a great bar to publication, and that brevity of style and conciseness of statement greatly facilitate early insertion.]

### LEGISLATION FOR MIDWIVES.

Sir,—Your correspondent "Z. Y. X." is pleased to be very witty at my expense, and also at the expense of midwives in general. But he will excuse my pointing out that he fails to grasp the real matter at issue in the Bill now happily, let us hope, defunct. In this particular "Z. Y. X." follows the example of all who have been talking about the Bill. No one seems to have grasped its real significance, or to have taken the trouble to set it before their eyes as it will work out in practice. Let us suppose that the Bill had passed, and has come into operation. The effect is this, that in every small village or thinly-populated district all over the kingdom there is one woman who has a monopoly of delivering her neighbours in labour. She may be an unscrupulous gossip, she may be rough in manner, she may for private or family reasons be distasteful to some of them. No matter, she is the only licensed midwife within reach, they are compelled to employ her. There are a hundred ways in which a midwife may be intensely objectionable to her patients, yet run no risk of losing her licence. It is true the mother may, as we have been somewhat brutally told, employ the parish doctor, but to do this they must obtain an order from the relieving officer, and must label themselves paupers. Now, sir, I maintain that to confer a monopoly of this description for the performance of an absolutely necessary function is such an interference with the liberty of the subject as has never been tolerated before in this country, and will not now be tolerated if only its inevitable tendency be clearly realised. Are we to compel our poorer neighbours by pains and penalties to employ just the one person who has gone through the ceremony of registration to assist at the natural process of delivering his wife in labour? If so it seems a little less than kind to leave the infant unprotected through the remainder of its life a prey to the wise woman, to the patent medicine vendor, the faith healer, and the quack. This monopoly which is to be set up in midwifery and conferred on ignorant women with the least possible smattering of their business is a monopoly which the most highly skilled medical attendant cannot command in matters of life and death. If I choose to employ a bone-setter to pull together my dislocated shoulder I expose him to no penalty, though I may expose myself to some risk. The most eminent surgeon in the county has no preference in law over my local handy man, and I have as full liberty to fee the one as the other. But my wife if she is too poor to pay the doctor's fees is delivered over *nolens volens* to the tender mercies of some village worthy, whose skill she may doubt and whose person she may dislike, because she is prohibited by law from paying a fee to her next door neighbour in whose skill and kindness she justly confides. I laid some stress in the articles which have appeared in your columns on the miserably low standard of midwifery which subsists in those countries which register their midwives. "Z. Y. X." has made no comment on this. It is one of those details which he says are too small to be noticed. But, sir, it is exactly this result which we may expect to follow the introduction of any class monopoly. So long as there is a healthy rivalry among the matrons of the district, who are prepared to earn a few shillings by assisting their neighbours in their confinements, so long will the work be, on the whole, well performed. Take away the stimulus of competition, and you take away a factor far more potent than any control which may be brought to bear through the operation of disciplinary clauses. There are hundreds of districts in which it is scarcely possible that there can be more than one midwife licensed. Indeed, it is by no means clear that the one registered midwife will always be forthcoming. The Bill makes absolutely no provision for districts in which no one desires to apply for a licence, and its promoters do not act wisely in merely reiterating that all this will come right by-and-by. In my opinion the penal clauses directed against unlicensed midwives are a gigantic mistake. Encourage these women to undergo instruction, and, if possible, to get training in a general hospital or workhouse infirmary. Confer licences on all who are properly qualified, and let special privileges go with the licence. All who seek to earn their living by this business will find themselves compelled to take out a licence by the overwhelming advantages accompanying them. But in the sparsely-populated districts where it is worth no one's while to settle down to this occupation, it is better to leave nature alone, and allow the mothers to help each other as before and take payment for what, after all, is a very laborious office, without introducing into these cottage homes an odious system of espionage, compulsion, and fines. Wherever competition arises it is safe to predict that the possessor of the Government licence, especially if that licence testifies to a real and solid qualification, will carry the public with her. I have trespassed too long on your space, and must omit any comment on the further observations of my critic.—Writer of "Legislation for Midwives."

